# Cognitive and behavioral clinical outcome assessments in children with developmental and epileptic encephalopathies: Issues and instruments

**DOI:** 10.1002/epi.70308

**Published:** 2026-06-05

**Authors:** Cinzia Correale, Nicola Specchio, Jenny Downs, J. Helen Cross, Simona Cappelletti, Andrea Palacio‐Navarro, Sam Geuens, Kette Valente, Andreas Brunklaus, Colin Reilly

**Affiliations:** ^1^ Center for Behavioral Sciences and Mental Health Istituto Superiore di Sanità Rome Italy; ^2^ Neurology, Epilepsy and Movement Disorders, Bambino Gesù Children's Hospital IRCCS, full member of European Reference Network EpiCARE Rome Italy; ^3^ University Hospitals KU Leuven Belgium; ^4^ Kids Research Institute Australia, Centre for Child Health Research University of Western Australia Perth Western Australia Australia; ^5^ Great Ormond Street Institute of Child Health University College London London UK; ^6^ Great Ormond Street Hospital for Children NHS Foundation Trust London UK; ^7^ Research Department Young Epilepsy Lingfield Surrey UK; ^8^ Epilepsy Unit, Pediatric Neurology Service, Hospital Sant Joan de Déu full member of European Reference Network EpiCARE Barcelona Spain; ^9^ Leuven Childhood Epilepsy Center, Leuven Brain Institute KU Leuven Leuven Belgium; ^10^ Department of Development and Regeneration KU Leuven Leuven Belgium; ^11^ Hospital das Clínicas Faculty of Medicine of the University of São Paulo Sao Paulo Brazil; ^12^ Faculty of Medicine University of Sao Paulo Sao Paulo Brazil; ^13^ School of Health and Wellbeing University of Glasgow Glasgow UK; ^14^ Paediatric Neurosciences Research Group Royal Hospital for Children Glasgow UK; ^15^ Department of Pediatrics, Institute of Clinical Sciences, Sahlgrenska Academy University of Gothenburg Gothenburg Sweden; ^16^ Queen Silvia Children's Hospital, Member of the ERN EpiCARE Sahlgrenska University Hospital Gothenburg Sweden

**Keywords:** clinical outcome assessment, clinical trial readiness, developmental, natural history

## Abstract

Children with developmental and epileptic encephalopathies (DEEs) face cognitive and behavioral challenges that may have a greater impact than seizures on their quality of life (QoL). The need to assess these nonseizure outcomes for evaluating treatments is increasingly recognized. Advances in genomic technologies have transformed the diagnostic landscape of rare genetic epilepsies, including DEEs, opening new opportunities for precision medicine. There is also growing interest in drug repurposing, identifying well‐tolerated medications for other indications for use in DEEs. Innovative trial designs and the systematic collection of prospective natural history data are essential, given the rarity and heterogeneity of DEEs. The selection of reliable, valid, and meaningful outcome measures of cognition and behavior is crucial for clinical trials and natural history studies. Commonly used tools for assessing cognition and adaptive behavior often exhibit floor effects and may fail to capture subtle, yet clinically significant, changes in functioning that are meaningful for children and their families. There is thus a need to explore a fuller range of clinical outcome assessments (COAs) and scoring methods that could be sensitive to change and suitable for use in rare disease populations. Assessing behavioral and emotional outcomes in children with DEEs is additionally challenging, as many assessment instruments have been developed and validated for use in children without intellectual disability. To fully realize the potential of precision medicine in the DEEs, a robust framework for outcomes assessment is required, one that incorporates sensitive, reliable, and meaningful COAs tailored to this population. Coordinated efforts to identify and adapt existing measures or develop new outcome tools will be crucial for advancing therapeutic strategies that genuinely improve QoL for children with DEEs. This article summarizes the issues faced when selecting outcome measures for DEE trials and reviews commonly used instruments for assessing cognition and behavior in children with DEEs.

**Key points**

- Difficulties with cognition and/or behavior may impact quality of life more than seizures in children with DEEs.
- Available measures of cognition and adaptive behavior frequently show floor effects in DEEs, limiting sensitivity to change.
- Alternative scoring methods better capture small but clinically meaningful developmental changes.
- Behavioral measures normed on ID populations are likely to be better than measures normed on typically developing populations.
- Caregiver‐anchored and functional outcomes are essential for trial readiness in DEEs.

## 1 INTRODUCTION

Developmental and epileptic encephalopathies (DEEs) are a group of severe epilepsies characterized by early onset and often drug‐resistant seizures and developmental slowing or regression.^1^, ^2^, ^3^ Developmental impairment arises from the combined effect of epileptic activity and underlying etiologies,^3^ resulting in substantial functional difficulties. Children with DEEs frequently present with impairments in cognitive functioning, adaptive behavior, and behaviors associated with neurodevelopmental disorders such as autism spectrum disorder (ASD) and attention‐deficit/hyperactivity disorder (ADHD), alongside emotional and behavioral problems.^4^, ^5^, ^6^ These difficulties profoundly impact health‐related quality of life (QoL) in children and contribute to substantial emotional, social, and occupational burden for caregivers and family members.^7^, ^8^, ^9^

Advances in genomic technologies have transformed the diagnostic landscape of epilepsy and opened new opportunities for precision medicine therapies.^10^, ^11^ Precision medicine aims to optimize treatment by targeting underlying disease mechanisms while considering the broader biological, psychological, and sociocultural context of the child.^12^ In DEEs, therapies targeting the underlying etiology hold promise for improving cognitive and behavioral outcomes in addition to seizure control.^10^ However, important challenges remain. Functional differences between variants within the same gene (e.g., gain vs. loss of function) and substantial phenotypic heterogeneity, even within apparently homogeneous monogenetic groups, complicate treatment selection and evaluation. Understanding phenotype–genotype variability is therefore essential to guide the selection of appropriate clinical outcome assessments (COAs) and ensure that treatments are suitable for individuals.^10^ In parallel, increasing interest has been given to drug repurposing strategies aimed at identifying existing, well‐tolerated drugs that may be effective in specific genetic epilepsies.^13^, ^14^, ^15^, ^16^ Given the rarity and heterogeneity of DEEs, natural history studies are crucial for clinical trial readiness, providing information on disease course, phenotypic variability, and trajectories over time.^17^ Such data are essential to contextualize treatment effects, inform inclusion criteria, and guide the selection of appropriate outcome measures.

A key challenge in both clinical trials and natural history studies in DEEs is the selection of appropriate outcome measures. Disease‐specific COAs for cognition and behavior are lacking for most DEEs. The involvement of clinicians and caregivers in COA development and evaluation has been widely recognized as essential to ensure that instruments are reliable, valid, and responsive for the target population, before being used as trial endpoints.^18^, ^19^ Accordingly, increasing efforts have focused on characterizing meaningful outcomes for DEEs.^19^, ^20^ These include studies exploring caregiver and clinician perspectives on meaningful changes in Dravet syndrome,^21^ investigations of alternative scoring methods to assess adaptive behavior in *SCN2A‐*related disorders,^22^, ^23^ and studies examining adaptive behavior and neurodevelopment in CDKL5 deficiency disorder.^24^ Other studies have evaluated commonly used cognitive and behavioral measures in DEEs, highlighting challenges related to validity, sensitivity to change, and interpretability in this population.^25^, ^26^

Despite the need to consider COAs in clinical trials and natural history studies, there has been a lack of comprehensive consideration of the issues and instruments relevant in cognitive and behavioral COAs in children with DEEs. In this paper, we discuss the methodological and interpretative challenges in assessing cognitive and behavioral outcomes in children with DEEs, review currently used instruments, and consider implications for the selection and reporting of cognitive and behavioral COAs in clinical trials and natural history studies. The primary focus of this paper is on cognitive and behavioral outcomes in children with DEEs, rather than on motor, language, or social outcomes. However, evaluating cognition and development in young children or in individuals with lower levels of intellectual functioning common in DEEs is often complicated by concurrent communication, motor, and social difficulties, which makes it challenging to isolate cognitive abilities. As a result, assessments for these children frequently incorporate measures of motor and communication skills alongside evaluations of cognition and adaptive behavior.

### 1.1 Principles for guiding the selection of COAs

There are established frameworks for evaluating the measurement properties of COAs, such as COSMIN (Consensus‐Based Standards for the Selection of Health Status Measurement Instruments),^27^ which aligns with US Food and Drug Administration (FDA) guidance on COAs to best inform clinical trial design.^18^ These emphasize the importance of validity, reliability, responsiveness, and interpretability in clinical measurement. Table 1 summarizes key measurement considerations, as applied to the selection of cognitive and behavioral outcome measures in DEEs.

**TABLE 1:** Measurement principles^18^, ^27^ to guide the selection of cognitive and behavioral outcome measures in DEEs.

Validity	Outcome measures should accurately measure what they are intended to assess, whether it is cognitive function, adaptive behavior, or behavioral and emotional development. The content of measures must be appropriate for the target population (e.g., children with DEEs).
Reliability	The outcome measure should produce consistent and reproducible results across different settings, raters, and time points when the clinical condition is stable.
Sensitivity to change	Outcome measures need to be able to detect clinically meaningful changes, even if these are small or can be demonstrated in subdomains. It is important to avoid "floor effects," which are common in severely affected individuals with DEEs.
Clinical meaningfulness	Outcomes should reflect priority aspects of health and functioning and be able to measure change that is meaningful to patients, families, and clinicians.
Feasibility and acceptability	The outcome measure should be practical to administer in terms of time, cost, and burden on patients and families.
Inclusion of clinician‐administered and caregiver‐reported outcomes	Capturing caregiver perspectives on functioning can complement clinician‐administered measures of cognition/development.
Standardization and harmonization	Administration of outcome measures should be standardized to allow comparisons across studies and facilitate meta‐analyses. Harmonizing COAs used across different DEEs, age groups, and abilities may allow for trial/study efficiencies and evaluations of comparative effectiveness.
Regulatory and stakeholder acceptance	Selected COAs should be acceptable to regulatory agencies, patient advocacy groups, and the wider clinical and research community.

Abbreviations: COA, clinical outcome assessment; DEE, developmental and epileptic encephalopathy.

### 1.2 Selecting instruments and interpreting data from cognitive and behavioral outcome measures in DEEs

Most individuals with DEEs have intellectual disability (ID). However, most instruments developed to assess cognitive functioning have been standardized on normative populations that included few children with ID, particularly children with moderate or severe/profound ID.^28^ As a result, many standard cognitive assessment tools are not suitable for use in populations with moderate or severe/profound ID, because they result in "floor effects" (see Box 1). This limitation reduces the ability of standard scores, such as intelligence or developmental quotients, to capture subtle variations in skills or longitudinal changes.^29^, ^30^

In addition, currently available instruments may not capture small but clinically meaningful improvements in areas such as attention, executive function, or language, making it difficult to define a "responder" (see Box 1). Standardized scores may also represent a suboptimal "unit of measurement" in DEEs. Rather than comparison with typically developing populations, interpretation may benefit from reference to disorder‐specific natural history data. In these situations, alternative metrics such as raw scores, age equivalents, or growth scale values (GSVs; see Table 2) may better capture within‐patient developmental trajectories and subtle changes over time. Practical challenges further complicate assessment. Many cognitive instruments rely on verbal, motor, and attentional demands that may be inappropriate for individuals with DEEs. As a result, these children may be assessed using instruments for younger children ("out of age testing"; see Box 1). Similarly, many behavioral and emotional assessment tools lack appropriate normative data for individuals with ID, particularly severe or profound ID.^30^ As a result, existing measures may fail to reflect gradual or domain‐specific changes that are highly valued by families and relevant for clinical trials.

**TABLE 2:** Definition, advantages, and disadvantages of scoring concepts in measures of cognition and adaptive behavior.

Concept	Definition	Advantages	Disadvantages
Standard scores	Scores transformed from raw data onto a common scale with a set mean and SD, enabling comparison across tests or groups (e.g., z‐scores).	Allow comparison across different tests and populations due to standardized scale. Account for variability in the population (mean and SD). Useful for identifying deviations from average performance.	Can be difficult to interpret without understanding the normative sample and scale. May obscure individual raw score differences by compressing or expanding data. May hinder the interpretation of change in subsequent follow‐up evaluations.
Raw scores	The original, untransformed scores obtained directly from a task or assessment before any statistical conversion or scaling.	Simple and direct measure of actual test performance. Easy to understand and calculate. It enables comparison of a patient's performance over time and the identification of neurodevelopmental regression.	Cannot be compared across different tests or populations without conversion. No context for whether a score is typical or atypical relative to a norm group.
GSVs	Scores that estimate a participant's developmental level relative to a version‐specific normative growth scale, often used to track progress over time.	Reflect developmental progress and changes over time within the same individual. Can be intuitive for tracking individual growth in longitudinal follow‐up.	Not always linear, making interpretation over time difficult. Can be misleading if used to compare between individuals or groups or across different test versions or cultural adaptations. Difficult to compare across subtests or test batteries
AEs	Scores that represent the average age at which a given raw score is typically observed in a normative sample, expressing performance in terms of developmental age. These scores express the performance expected from that age window.	Translate test performance into a developmental age that is easy to understand. Useful for communicating results to caregivers and nonspecialists. Can support longitudinal within‐patient interpretation, including estimation of developmental pace over time.	Anchored to version‐specific normative samples. May imply unrealistic precision about developmental level and overestimate the performance. May not reflect actual functional ability and can be misinterpreted as chronological age. Not comparable across measures, versions, or normative samples.

Abbreviations: AE, age equivalent; GSV, growth scale value.

BOX 1 **Considerations when selecting COAs for cognition and behavior in DEEs.**

**Floor Effects:** Many commonly used cognitive assessment tools are limited by floor effects^31^, ^32^, ^33^ and may fail to detect subtle, yet clinically meaningful, improvements in children with DEEs.^34^ Because normative distributions are derived from typically developing children, the lower tail of the scale provides insufficient gradation to detect subtle differences that can be clinically meaningful for caregivers and relevant for evaluating treatment effects in DEEs. In low‐functioning children, subtest scores often cluster at the floor, preventing distinction between degrees of impairment. The simplest items on the widely used Wechsler tests (e.g., pointing, naming, following instructions) presume a baseline developmental age of approximately 3 years. Children with significant delays (developmental age < 3 years) may be unable to meaningfully engage with the tasks. For example, a child with a developmental age of 2 years may obtain the minimum score across several subtests of the Wechsler scales regardless of whether their true developmental level corresponds to an intelligence quotient of 20 or 50. In addition, standard scores on Wechsler tests do not go below 40. These limitations have been demonstrated empirically. In the BUTTERFLY study of children and adolescents with Dravet syndrome, standard cognitive measures, including Wechsler scales and the Bayley Scales of Infant and Toddler Development, showed pronounced floor effects and limited ability to capture change over time, despite clinical evidence of variability or progression in functional abilities.^35^ Similarly, commonly used adaptive behavior measures such as the Adaptive Behavior Assessment System, Third Edition (ABAS‐3) and Vineland Adaptive Behavior Scales, Third Edition (VABS‐III) also exhibit floor effects in children with profound ID.^36^, ^37^

**Out of Age Testing:** Administering a test designed for a specific age group to individuals outside the intended age range may affect validity and interpretability, as these instruments are standardized on age‐specific normative samples.^38^ This practice can lead to misinterpretation by either overidentifying or underidentifying developmental concerns. Whenever possible, clinicians assessing children with DEEs should prioritize tools validated for the child's chronological age to ensure reliable and clinically meaningful results.

However, in the absence of age‐appropriate instruments, a scenario not uncommon in DEEs, carefully interpreted out of age testing may still provide valuable information. For example, a school‐aged child with severe/profound ID may only be able to engage with tasks designed for toddlers, such as simple object recognition or imitation tasks. Tasks developed for younger developmental age can help assess basic abilities, such as approach to objects, eye–hand coordination, basic visual recognition, and understanding simple instructions. With appropriate adaptation, repeated administration may help document small but meaningful changes over time.

**Definition of a Responder:** Assessment challenges in DEEs and slow, uneven developmental trajectories influence how clinical response should be conceptualized. Traditional responder definitions based on fixed thresholds on standardized tests may be insufficient, as small within‐patient changes can be meaningful even when standardized scores show minimal or no improvement.^39^, ^40^, ^41^ Patient‐centered work suggests that responders in DEEs may instead be identified through subtle functional improvements, for example, increases in communication of needs, understanding instructions, or engagement in daily routines, which caregivers consistently describe as meaningful.^19^, ^41^, ^42^ Responder definitions may be more informative when anchored to individual contexts rather than normative expectations, recognizing stabilization, reemergence of microskills, or modest shifts in participation as potential indicators of benefit. These considerations are consistent with FDA Patient‐Focus Drug Development (PFDD) principles, which emphasize the identification of outcomes that are meaningful to patients and caregivers and interpretable in daily life. In DEEs, such principles have been articulated through FDA‐led PFDD initiatives, including disease‐specific meetings in Lennox–Gastaut syndrome (https://www.lgsfoundation.org/patient‐focused‐drug‐development‐meeting/, accessed January 15, 2026), *CDKL5* deficiency disorder (https://www.cdkl5.com/wp‐content/uploads/2023/05/CDD‐VoP‐REPORT.pdf, accessed January 15, 2024), and *KCNT1*epilepsy (https://static1.squarespace.com/static/5f0d4aee86be2a6c00460b94/t/66563a8cfdade62a6990fb36/1716927117355/KCNT1+PLS+Summary.final.pdf).

### 1.3 Scoring and interpreting COAs for cognition and adaptive behavior in DEEs

Beyond the selection of valid outcome measures in DEEs, there are a number of pertinent concepts in relation to scoring and interpreting scores derived from assessment instruments (Table 2). It is important to note that not all commonly used instruments in children with DEEs provide the same scoring possibilities, and these are noted in Table 3.

**TABLE 3:** Commonly used instruments for assessing cognition/development and adaptive behavior in children with DEEs.

Instrument^a^	Domain assessed	Age	Range of standard scores	Scoring	Comment
WISC‐V^43^ WPPSI‐IV^44^ WAIS‐IV^45^	Cognition including FSIQ and 15 subscales	2.6 years to adulthood	40–160	Raw, standard	Floor effects. Not suitable for children with severe/profound ID. Moderate ID questionable.
Bayley‐IV^46^	Cognition, communication (receptive and expressive), and motor skills (gross and fine)	0–42 months	55–160	Raw, standard, GSV, AE	Must be used out of age range for children older than 42 months. No composite DQ/IQ score.
GSMD‐III^47^	Development (5 subscales: foundations of learning, language and communication, eye and hand coordination, personal–social–emotional, and gross motor) including composite score, DQ	0–72 months	40–160	Raw, standard, AE	Must be used out of age range for children older than 72 months.
MSEL^48^	Development (5 subscales: gross motor, visual reception, fine motor, receptive language, and expressive language) including composite score, early learning composite	0–68 months	49–155	Raw, standard, AE	Must be used out of age range for children older than 68 months.
VABS‐III^49^	Adaptive behavior in 4 domains: communication, daily living, community, and motor; includes adaptive behavior composite	0–89 years	20–160	Raw, standard, GSV, AE	Lowest standard score of 20.
ABAS‐3^50^	Adaptive behavior in 3 domains—conceptual, social, and practical—and a general adaptive composite	0–89 years	40–160	Raw, standard, AE	Lowest standard score of 40.
DP‐4^51^	Development across 5 domains: physical, adaptive behavior, social–emotional, cognitive, communication; includes general development score	0–21 years	40–160	Raw, standard, AE, GSV	Caregiver report measure; useful for broad developmental profiling and longitudinal follow‐up. Lowest standard score of 40.

Abbreviations: ABAS‐3, Adaptive Behavior Assessment System, Third Edition; AE, age equivalent; Bayley‐IV, Bayley Scales of Infant and Toddler Development, Fourth Edition; DP‐4, Developmental Profile, Fourth Edition; DQ, developmental quotient; FSIQ, full scale IQ; GSMD‐III, Griffiths Scales of Mental Development, Third Edition; GSV, growth scale value; ID, intellectual disability; IQ, intelligence quotient; MSEL, Mullen Scales of Early Learning; VABS‐III, Vineland Adaptive Behavior Scale, Third Edition; WAIS‐IV, Wechsler Adult Intelligence Scale, Fourth Edition; WISC‐V, Wechsler Intelligence Scale for Children, Fifth Edition; WPPSI‐IV, Wechsler Preschool and Primary Scale of Intelligence, Fourth Edition.

^a^ Data reported on latest edition and English language version.

### 1.4 Instruments for measuring development/cognition and adaptive behavior in DEEs

Commonly used measures for assessing cognition/development and adaptive behavior in children with DEEs and their characteristics are shown in Table 3.

### 1.5 Assessing behavior and emotions in DEEs

Behavioral and emotional challenges are common in children with DEEs,^25^, ^52^ creating a need for reliable assessment tools. Checklists commonly used in pediatric populations such as the Strengths and Difficulties Questionnaire (SDQ)^53^ and the Child Behavior Checklist (CBCL)^54^ are widely validated in typically developing populations and may also be informative in children with mild ID.^55^, ^56^ However, these instruments are generally not suitable for children with moderate to severe/profound ID,^5^ which is common in DEEs. Both the SDQ and CBCL lack ID‐specific norms, and thus these tools may over‐ or underestimate behavioral problems.^57^

Common behaviors in ID (e.g., repetitive actions, limited verbal skills, sensory sensitivities) risk being misinterpreted as symptoms of ASD, anxiety, ADHD, or conduct problems or alternatively being attributed to ID alone, leading to diagnostic overshadowing.^58^ Although aggressiveness is frequently reported in DEEs, commonly used behavior checklists often conceptualize aggression as intentional behavior with a willingness to cause harm. This conceptualization does not adequately capture the behavioral dysregulation typically observed in children with ID and DEEs, where behaviors are more often related to impaired self‐regulation, communication difficulties, or sensory reactivity rather than deliberate intent. Furthermore, some checklists include items that rely on motor activity or physical capacities (e.g., restlessness, running, climbing, or fidgeting), which may be substantially affected by the neurological and motor impairments frequently found with DEEs, thereby further complicating interpretation of scores. Informant ratings can also be influenced by expectations based on age rather than developmental level. Lastly, some items on these scales may feel irrelevant or inappropriate for children with DEEs as they require a certain level of cognitive (e.g., stealing) and/or verbal (e.g. lying) ability that many children with DEEs do not have. Asking about these items may undermine caregiver trust in the assessment process and lead to frustration as well as not providing valid results.

In light of these limitations, employing behavioral measures specifically developed for individuals with ID, which are often better aligned with the developmental profiles and levels in DEEs, may be preferable. Some examples include the following:

- Developmental Behavior Checklist (DBC)^59^—validated for children and adolescents with ID.
- Aberrant Behavior Checklist (ABC)^60^—widely used in individuals with ID and other populations with a neurodevelopmental disorder.
- Behavior Problems Inventory (BPI)^61^—specifically designed to assess frequency and severity of self‐injurious, stereotyped, and aggressive/destructive behaviors in individuals with ID and severe developmental disabilities.

The ABC has been previously employed in a study of children with etiology‐heterogeneous DEEs,^25^ whereas the DBC has been used in the study of behavior in Dravet syndrome.^62^ The BPI is particularly useful for capturing core maladaptive behaviors that are highly prevalent and clinically relevant in children with DEEs. Table 4 provides an overview of the characteristics of instruments discussed.

**TABLE 4:** Commonly used instruments for measuring behavior and emotions in the pediatric population and their utility in DEEs.

Instrument	Age	Versions	Domains	Utility in ID populations
SDQ^53^	2–17 years	Caregiver, teacher, and self‐ report	5 subscales and total score	Not designed for ID populations; less likely to be useful in moderate, severe, or profound ID.
CBCL^54^	18 months to 18 years; ABCL is adult version	Caregiver, youth self‐ report, and teacher forms	8 subscales, total problems, and internalizing/externalizing subscales; DSM‐oriented scales	Not designed for ID populations; less likely to be useful in moderate, severe or profound ID.
DBC^59^	4–18 years	Caregiver, teacher versions	5 subscales and total score	Designed for ID population.
ABC^60^	5+ years	Caregiver	5 subscales	Designed for ID population.
BPI^61^	Children, adolescents, and adults	Caregiver report	Maladaptive behaviors: self‐injurious behavior, stereotyped behavior, aggressive/destructive behavior	Designed for ID populations; focuses on core maladaptive behaviors; limited coverage of emotional/internalizing domains

Abbreviations: ABC, Aberrant Behavior Checklist; ABCL, Adult Behavior Checklist; BPI, Behavior Problems Inventory; CBCL, Child Behavior Checklist; DBC, Developmental Behavior Checklist; DEE, developmental and epileptic encephalopathy; DSM, Diagnostic and Statistical Manual; ID, intellectual disability; SDQ, Strength and Difficulties Questionnaire.

In addition to assessing broad‐based behavioral and emotional functioning, it may also be important to consider behaviors associated with ADHD and ASD, as these are common in DEEs^4^, ^63^ and may significantly impair functioning and quality of life. Standard diagnostic and screening tools for ASD, although widely used and validated across a broad range of development abilities, may demonstrate reduced reliability and validity when applied to children with severe/profound ID,^64^ common in DEEs, and thus may not be suitable as outcome measures in clinical trials and natural history studies. For example, the Autism Diagnostic Observation Schedule, Second Edition,^65^ often considered a gold‐standard observational tool for ASD, has less validity in minimally verbal children.^66^ At developmental levels below 18 months, distinguishing between behaviors stemming from ID and those indicative of ASD becomes particularly challenging.^67^ In relation to symptoms of ADHD, many behaviors overlap with developmentally typical behaviors in young or developmentally delayed children. High activity, short attention span, impulsivity, and poorer compliance are expected at certain developmental stages or intellectual levels. In some children with DEEs, these behaviors are developmentally appropriate, not pathological. There are currently no validated ADHD‐specific norms for individuals with severe/profound ID, and commonly used rating scales (i.e., Conners Ratings Scales,^68^ Swanson, Nolan, and Pelham Teacher and Parent Rating Scale, Version IV,^69^ Vanderbilt ADHD Diagnostic Rating Scale^70^) are normed on typically developing children or those with mild ID. Without ID‐specific norms or items, changes in scores can be misleading, and/or the measures may not detect meaningful behavioral changes in children with moderate‐to‐profound ID.

Given these limitations, ID‐specific behavioral assessment tools that include ASD or ADHD subscales may provide a useful way to evaluate these symptoms in this population. The DBC^59^ is normed for children with ID and includes a 29‐item autism subscale, which has been used in a study of children with Dravet syndrome.^63^ The DBC also includes a subscale for measuring hyperactivity and inattention. Additionally, the ABC^60^ includes a hyperactivity/noncompliance subscale. As these instruments are normed in individuals with ID, they are more likely to be sensitive to changes in symptoms associated with ASD and ADHD in DEEs, although further data are needed to confirm this.

### 1.6 Practical examples of alternative scoring methods in COAs of cognition and adaptive behavior in DEEs

**FIGURE 1:**
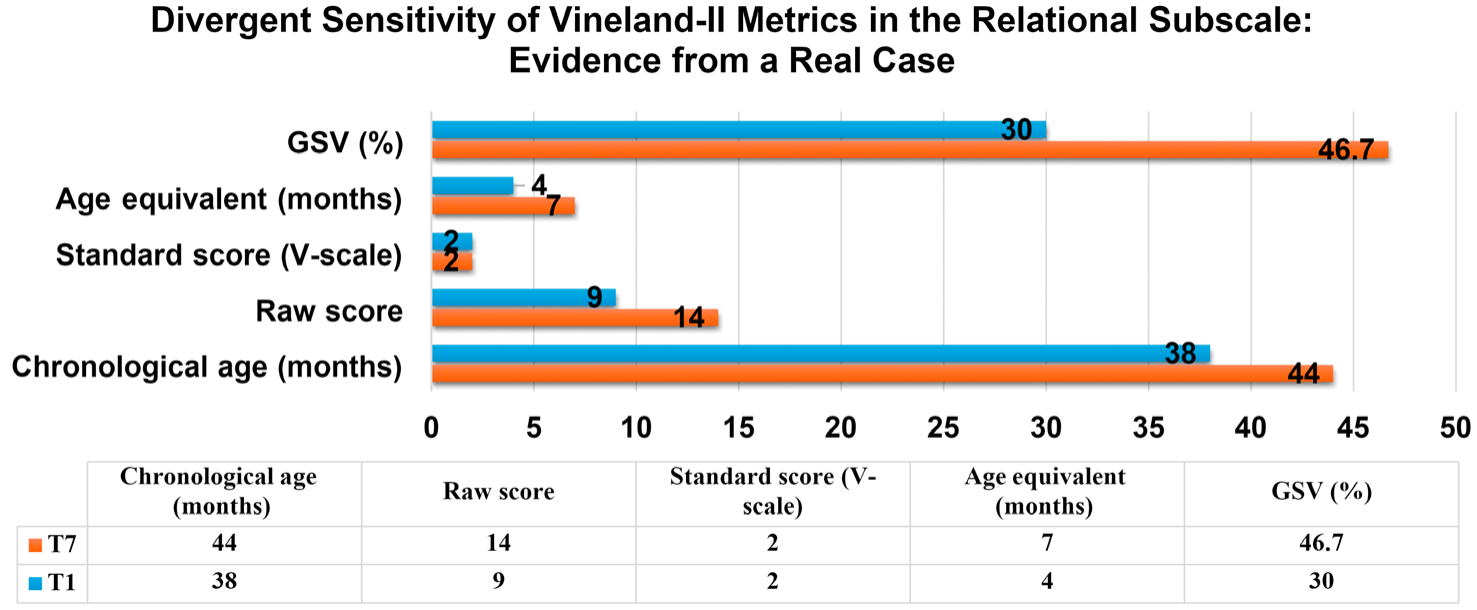
Comparison of Vineland‐II metrics (raw score, age equivalent, growth scale value [GSV], and V‐scale standard score) between baseline (T1, chronological age = 38 months) and follow‐up (T7, chronological age = 44 months) for the interpersonal relationships domain in a child with developmental and epileptic encephalopathy due to a pathogenic *HUWE1* variant. Raw score, age equivalent, and GSV increase over time, whereas the V‐scale remains unchanged, illustrating the impact of floor effects in norm‐reference scores.

The examples in Box 2 describe practical applications of alternative methods of scoring in studies of children with DEEs. They illustrate how alternative scoring strategies can enhance sensitivity to meaningful change when standardized scores are low, in line with FDA PFDD guidance on fit‐for‐purpose outcome assessments. Additionally, a real‐world example of an individual patient is illustrated in Figure 1. The figure compares four VABS‐II^71^ metrics (raw score, age equivalent, GSV, and standard score/V‐scale) between two assessment time points in a child with DEE caused by a pathogenic *HUWE1* variant. Following the introduction of an investigational antiseizure treatment and subsequent enrollment in a clinical trial, the child underwent regular follow‐up assessments of adaptive functioning. During the retest interval (38–44 months chronological age), caregivers reported progressive developmental improvements, particularly in motor engagement and environmental responsiveness, together with a marked reduction in spasm frequency (from approximately 70 to ~10 episodes/day). Consistent with these reports, raw score, age equivalent, and GSV all increased over time, indicating measurable developmental gains. In contrast, the V‐scale (norm‐referenced standard score) remains unchanged, illustrating how floor effects may obscure meaningful clinical improvements when relying solely on norm‐based metrics.

BOX 2 **Examples of alternative scoring methods for COAs measuring cognition and behavior in DEEs.**

**Age Equivalents:** Pietrafusa et al.^72^ employed the Griffiths Mental Development Scales^47^ and interpreted results using developmental quotients and age equivalents in a study evaluating fenfluramine in infants with Dravet syndrome younger than 2 years. This allowed for the demonstration of stability or gains in specific subdomains, even when standardized scores remained unchanged or declined. Age equivalents do not benchmark performance against neurotypical peers but captured within‐patient developmental level and progression. This prevents misinterpretation of apparent “regression” relative to normative trajectories and instead highlights the pace of progression. This approach aligns with recommendations to adapt outcome scoring when standardization obscures clinically relevant change.^18^

**GSVs:** The CANDID (Clinical Assessment of Neurodevelopmental Measures in CDD) longitudinal study of individuals with *CDKL5* deficiency disorder (CDD)^24^ used GSVs from the Vineland‐III^49^ and Bayley‐4.^46^ Use of GSVs highlighted skill acquisition with age in the Bayley‐4 motor skills domain, as well as in the Vineland‐III receptive language, interpersonal relationships, and fine motor subdomains. These changes in performance would not have been demonstrated if standard scores had been employed.

**Goal Attainment Scaling (GAS)/Personalized Endpoints:** In the *SCN2A* Clinical Trials Readiness program, Berg et al.^22^ and Sevinc et al.^20^ explored GAS to overcome heterogeneity and avoid floor effects. GAS allowed individualized, observable goals to be defined with caregivers and then rated on a standardized scale. This approach aims to capture progress of single targeted skills where gold‐standard instruments lack sensitivity to changes in that skill. The method is well aligned with FDA PFDD principles, which emphasize outcome measures that are relevant and meaningful to families. Advantages include high sensitivity and personalization, whereas limitations include the need for a rigorous approach to goal‐setting for consistency and standardized scoring to ensure comparability across participants.

**Caregiver‐Anchored Meaningful Change:** Downs et al.^19^ applied a mixed‐methods design to identify what caregivers of children with DEEs consider to be meaningful change in functional abilities. Their findings underscored the gap between standardized test scores and lived experiences, where small changes in the level of skills that are poorly captured by standardized scores may be important and meaningful for families, because they could improve functioning in both the developmental domain and other domains (e.g., better sleep or mental health, more consistent response to name, easier activities of daily living). The study suggested that outcomes interpreted using caregiver‐defined anchors could reflect real‐world benefit. This approach is explicitly encouraged by FDA PFDD guidance, which highlights the importance of integrating patient and caregiver experience data into endpoint definition.

### 1.7 Other issues to consider in using COAs in children with DEEs

#### 1.7.1 Demographics, culture, and language

It is important to consider how a COA performs across the full range of participants to be enrolled in a trial/study, including factors such as age, race and ethnicity, and gender.^18^ Drug development programs for DEEs are often multinational; therefore, it is necessary to consider how language, cultural differences, and local customs may affect the relevance, consistency of responses, and interpretability of COAs across different study sites. Furthermore, outcome measures are not available and/or validated in all languages, which may limit their relevance and thus restrict participation and reduce the representativeness of the research. Moreover, the expectations and lived experiences of caregivers vary across cultures, gender, and family environments, influencing the interpretation of behavioral or adaptive changes and, consequently, what they consider a meaningful response. These contextual differences reinforce the need to view responder definitions not as fixed thresholds but as interpretive frameworks that must account for diverse developmental baselines and sociocultural contexts.^73^ Many cognitive and behavioral measures are normed on samples drawn from specific countries or cultural contexts, meaning that normative data from one population may not be directly applicable to participants from other countries.^74^

#### 1.7.2 Stage of the disease

The aspects of the disease that are meaningful to patients and caregivers, and that could be assessed to evaluate treatment effectiveness, may vary across different stages of the disease and varying levels of severity. In DEEs, practical considerations such as number of available patients may necessitate enrolling participants representing a broad range of disease stages, for example, with varying severity of symptoms. It is important to recognize that the validity, sensitivity, reliability, and interpretability of an endpoint may differ between patients with mild, severe, or even progressive disease. Different stages of the same disease can differ in the presence or severity of key characteristics, which may impact aspects of clinical investigation design—such as statistical power—and influence how endpoints are interpreted. Given these complexities, it is important to consider patient population characteristics and enrollment criteria when selecting COAs.

#### 1.7.3 Follow‐up period

The follow‐up period in studies should be long enough to ensure that an effect can be measured. Children with DEEs often live with exacerbations of seizure activity, gastrointestinal function, and poor sleep, which can affect functioning and performance during formal assessment. For some children and families, achieving stability as opposed to developmental progress may be seen as meaningful by caregivers.^41^ Fluctuating medical status impacts development in DEEs,^52^ and follow‐up assessment should consider multiple time points and long‐term follow‐up to assess developmental trajectories accurately. Choosing appropriate follow‐up periods in DEE studies may be challenging, as developmental trajectories across different etiologies are still insufficiently characterized and can vary widely in both pace and pattern.

Current knowledge suggests that considering the typical rate of developmental change described in each DEE may help inform expectations, even if it cannot yet guide exact timing. For example, in Dravet syndrome, longitudinal observational data from the BUTTERFLY study suggest that over 12–24 months, changes in adaptive functioning are small and domain‐specific (e.g., receptive communication and coping skills show detectable improvement, whereas expressive communication, interpersonal, gross motor, fine motor show little change), supporting the need for longer follow‐up and sensitive score metrics when targeting nonseizure outcomes.^75^ In parallel, qualitative work indicates that even 1–3 Vineland‐III raw score points (≈2–3 GSV points) may be considered meaningful by caregivers/clinicians, reinforcing the value of selecting endpoints that can capture small but clinically relevant gains.^21^ When natural‐history information is limited—which is common in many DEEs—it may be more informative to allow follow‐up periods that are sufficiently long (12+ months) to observe subtle developmental shifts, rather than scheduling retests at very short intervals, where changes may be difficult to interpret. Slightly longer intervals may increase the likelihood of detecting stabilization after a period of decline, small functional gains, or changes in developmental trajectory, outcomes that families and clinicians often consider meaningful, even when not reflected in large numerical shifts.^21^ This perspective is consistent with broader PFDD principles emphasizing that outcome assessment strategies should aim to maximize interpretability and capture change that is meaningful to families.^76^ Allowing sufficient time for within‐patient changes to emerge may support a more reliable interpretation of cognitive and behavioral outcomes in DEE clinical and research settings.

#### 1.7.4 Comorbid sensory and motor impairments and implications for neuropsychological assessment in DEEs

Some children with DEEs present with severe/profound ID accompanied by significant motor, visual, hearing, and communication impairments.^52^ These impairments can substantially limit the validity and interpretability of COAs, as performance on standardized tasks is often constrained by sensory, motor, or communicative barriers rather than reflecting the underlying cognitive or behavioral constructs. Thus, evidence derived from verbal children or those without major motor or sensory impairments may not be directly transferable to nonverbal individuals or to those with severe/profound ID and multiple comorbidities.^77^ Failure to account for these factors risks underestimating abilities, misclassifying severity, and misinterpreting change over time. Basic motor abilities, such as pointing or controlled eye gaze, are often prerequisites for tasks assessing memory, visuospatial skills, or receptive language; when these abilities are compromised, conventional testing may be inappropriate, as highlighted in the assessment of nonseizure outcomes in Lennox–Gastaut syndrome within the ESTEL (Electrical Stimulation of Thalamus for Epilepsy of Lennox–Gastaut Phenotype) trial.^78^ Finally, motor and speech impairments associated with DEEs may mask underlying cognitive abilities due to assumptions linking physical disability with low cognitive potential. Evidence from populations with severe motor impairment, such as cerebral palsy, indicates that adapted assessment strategies and assistive approaches can reduce motor and speech demands and allow a more accurate appraisal of functional abilities.^79^

#### 1.7.5 Ensuring quality administration of COAs and data management in DEEs

Providing detailed protocols and standardized training for assessment procedures can enhance accuracy, as well as intrarater, interrater, and test–retest reliability. Risk of bias can also be reduced by having evaluations conducted by blinded assessors who are not involved in other aspects of the trial and do not know whether the trial participant is receiving the treatment or placebo. In studies with small sample sizes, it is important to capture as much information as possible from each participant's COA. Dichotomizing continuous measures into simple categories (e.g., converting a continuous variable into a binary outcome) can lead to a loss of valuable information and should be avoided whenever possible. Transparency in reporting adaptations, scoring decisions, and handling of floor effects can further support reproducibility and regulatory confidence. Finally, integrating caregiver‐reported information or qualitative anchors, when methodologically appropriate, can enhance interpretability by providing context that numerical scores alone may not capture.^76^

In studies involving children with DEEs, missing data and increased variability should be anticipated rather than treated as exceptional. Illness, fatigue, behavioral dysregulation, and fluctuating medical status frequently interfere with task completion, particularly in longitudinal designs. Strategies such as scheduling assessments at optimal times of day, using flexible testing sessions with planned breaks, and involving examiners experienced in working with ID/DEE populations can help minimize nondevelopmental sources of variability and improve the reliability of assessments. Data‐driven strategies for handling incomplete assessments are also essential. Research on quality‐of‐life measures in DEEs has demonstrated that simulation‐based approaches using complete datasets can inform the development of empirically derived missing‐data rules, allowing valid score interpretation even when some items are missing.^80^ Such approaches support the use of measures that are tolerant to real‐world assessment challenges while preserving interpretability.

## 2 DISCUSSION

Measuring changes in cognition using traditional metrics, such as standard scores, poses many challenges for children with DEEs. Alternative scoring methods, such as raw scores, GSV, and age equivalents, enhance sensitivity and reduce the risk of misinterpreting apparent decline, plateau, or lack of progress when judged against neurotypical norms. These alternative scoring approaches instead situate outcomes within the expected natural history of the disorder, or when such data are unavailable, allow the child to serve as their own control to evaluate treatment impact on developmental progress, decline, or maintenance. Emphasizing trajectory and rate of developmental change is more informative than benchmarking against normative peers, as it captures whether a child is progressing better, worse, or as expected for their specific condition. Incorporating caregiver perspectives and individualized endpoints (e.g., GAS) ensures that outcomes reflect what families perceive as clinically meaningful in daily life, complementing quantitative metrics. Triangulating methods, combining adapted scoring from gold‐standard tools with caregiver‐reported outcomes and clinician observations, strengthens interpretability and aligns assessment with FDA PFDD guidance on fit‐for‐purpose measures. Using instruments normed for children with ID will ensure that behavioral and emotional outcomes are valid.

The key points for clinicians and researchers to consider when assessing children with DEEs, including the use of cognitive and behavioral COAs in clinical trials and natural history studies, are summarized in Box 3.

BOX 3 **Summary of key points in considering cognition and behavior in clinical practice and trials and natural history studies of children with DEEs.**

**Use Multimethod, Multi‐informant Assessment:**

- Combine caregiver reports, clinical observations, and direct testing when feasible.
- Gather input from multiple caregivers or professionals to reduce bias and increase reliability.

**Focus on Functional and Meaningful Outcomes:**

- Include adaptive functioning, QoL, communication, and behavior and emotional functioning instead of relying on only cognitive/developmental scores.
- Define clear, realistic, and clinically meaningful endpoints that reflect changes important to families and clinicians.

**In Direct Assessment of a Child With a DEE, Try to Reduce Behavioral and Attention Challenges**:

- Schedule assessments at optimal times of day.
- Use breaks and flexible testing sessions to reduce fatigue or distress.
- Employ experienced examiners trained in working with ID populations.

**Ensure Consistency of Administration and Scoring and Support Caregivers:**

- Provide clear instructions and support for informants completing questionnaires.
- Use interviews or guided administration to ensure accurate data.
- Brief home videos capturing key aspects of daily routines (e.g., communication attempts, mobility, feeding, sleep–wake transitions) may provide complementary qualitative information, particularly when direct assessment is not feasible or performance is likely to be variable.

**Plan for Missing Data and Variability in Clinical Trials and Natural History Studies:**

- Expect missing data due to participant noncompliance or illness.
- Use statistical methods robust to missing data (e.g., mixed models, imputation).
- Consider longer follow‐up to capture changes.

## 3 CONCLUSIONS

Assessing cognitive and behavioral outcomes in natural history studies and clinical trials in DEEs can present significant challenges. The selection of developmentally appropriate and sensitive measures is critical. Carefully planning assessment procedures is also necessary to reduce the potential for behavioral challenges and missing data. Standard scores will often provide limited and/or misleading data, and thus interpretation of outcomes should use appropriate scoring metrics and integrate with qualitative information to elicit a comprehensive picture. There remains also a clear need for further validation work to determine how well existing cognitive and behavioral measures perform in children with DEEs. Finally, the development of consistent standards for selection, interpretation, and reporting of nonseizure outcomes in DEE studies will be essential to support comparability, reproducibility, and interpretability across investigations.

## AUTHOR CONTRIBUTIONS

Cinzia Correale and Colin Reilly conceptualized the study, led its design, coordinated the work, and drafted the manuscript. Jenny Downs and Kette Valente made a substantial contribution to the conceptual framework and interpretation of the literature and critically revised the manuscript for important intellectual content. Nicola Specchio, J. Helen Cross, and Andreas Brunklaus provided senior clinical and scientific expertise, and critically revised the manuscript for intellectual content. Simona Cappelletti, Andrea Palacio‐Navarro, and Sam Geuens contributed to the interpretation of the data and revised the manuscript for intellectual content. All authors reviewed and approved the final version of the manuscript and agree to be accountable for all aspects of the work in accordance with International Committee of Medical Journal Editors criteria.

## CONFLICT OF INTEREST STATEMENT

N.S. has served on scientific advisory boards for Jazz Pharmaceuticals, BioMarin, Arvelle, Marinus, Takeda, UCB, and Stoke Therapeutics; has received speaker honoraria from Jazz Pharmaceuticals, UCB, Angelini, BioMarin, and LivaNova; and has served as an investigator for Marinus, BioMarin, UCB, Jazz Pharmaceuticals, Roche. J.D. has received funding for consultancy for Marinus, Ultragenyx, Acadia, Avexis, Orion, Takeda, Neurogene, Neurocrine, Taysha, and UCB; clinical trials with Anavex and Newron; and consulting/advisory board membership for *SCN2A* Australia. All remuneration has been made to her department. J.H.C has acted as an investigator for studies with Jazz Pharmaceuticals/GW Pharmaceuticals, Stoke Therapeutics, UCB/Zogenix, Ultragenyx, Encoded, Epigenyx, and Lundbeck; has been a speaker and has served on advisory boards for Biocodex, Jazz Pharmaceuticals, Nutricia, Stoke Therapeutics, and UCB (all remuneration has been paid to her department); holds an endowed chair at the University College of London Great Ormond Street Institute of Child Health; has received grants from the National Institute for Health and Care Research (NIHR), the Engineering and Physical Sciences Research Council, the Great Ormond Street Hospital for Children Charity, LifeArc, and Epilepsy Institute UK; and her research is supported by the NIHR Great Ormond Street Hospital Biomedical Research Centre. K.V. is Associate Editor of *Epilepsy & Behavior* and *Epilepsia*. She is currently collaborating on research with Ulysses Neuroscience, supported by the LouLou Foundation. She has served as a consultant or received honoraria for lectures from Lundbeck‐Longboard, Biocodex, UCB Pharma, Takeda, Praxis, Abbott, PTC, United Medical, and Latin‐American Pharmaceutical Industry (ACHE, Prati‐Donaduzzi, Eurofarma, EMS, and Zodiac Pharmaceuticals). She has been the principal investigator for clinical trials for Lundbeck‐Longboard, Takeda, Praxis, and the Latin‐American Pharmaceutical Industry (Prati‐Donaduzzi Pharmaceuticals). These activities are unrelated to this work. A.B. has received honoraria for presenting at educational events, serving on advisory boards, and consultancy work for Biocodex, Jazz Pharmaceuticals, Encoded Therapeutics, Servier, Stoke Therapeutics, and UCB. C.C, S.C, A.P.‐N, S.G., and C.R report no conflicts of interest. We confirm that we have read the Journal's position on issues involved in ethical publication and affirm that this report is consistent with those guidelines.
